# Clinical outcome of empiric antimicrobial therapy of bacteremia due to extended-spectrum beta-lactamase producing *Escherichia coli *and *Klebsiella pneumoniae*

**DOI:** 10.1186/1756-0500-3-116

**Published:** 2010-04-27

**Authors:** Vikas P Chaubey, Johann DD Pitout, Bruce Dalton, Terry Ross, Deirdre L Church, Daniel B Gregson, Kevin B Laupland

**Affiliations:** 1 Department of Medicine, University of Calgary, Calgary, Canada; 2 Department of Pathology and Laboratory Medicine, University of Calgary, Calgary, Canada; 3 Department of Critical Care Medicine, University of Calgary, Calgary, Canada; 4 Department of Microbiology and Infectious Diseases, University of Calgary, Calgary, Canada; 5 Calgary Laboratory Services, Alberta Health Services, Calgary, Canada; 6 Centre for Anti-microbial Resistance, Alberta Health Services, Calgary, Canada; 7 Department of Pharmacy Services, Alberta Health Services, Calgary, Canada

## Abstract

**Background:**

Prompt administration of adequate empiric antimicrobial therapy is a major determinant influencing the outcome of serious infections. The objective of this study was to describe empiric antimicrobial therapy employed and assess its effect on the outcome of patients bacteremic with extended-spectrum beta-lactamase (ESBL) producing *Escherichia coli *and *Klebsiella pneumoniae*.

**Findings:**

A retrospective surveillance study of all patients with bacteremias caused by ESBL-producing *E. coli *and *K. pneumoniae *(EK-ESBL) from 2000-2007 in the Calgary Health Region was conducted. Data were available for 79 episodes of bacteremia among 76 patients. Forty-four (56%) were male, the median age was 70.0 yrs [interquartile range (IQR) 60.6-70.1 yrs], and 72 (91%) episodes were *E. coli*. Seventy-four episodes (94%) were treated with empiric therapy within the first 48 hours. A non-statistically significant increased mortality occurred in those treated empirically with a beta-lactam/beta-lactamase inhibitor combination (6/16; 38% vs. 10/53; 18%; p = 0.063) while empiric carbapenem therapy was associated with lower mortality (0/10 died vs. 16/53 (30%), p = 0.089). Only 42 (53%) episodes received adequate therapy within the first 48 hours. The median time to first adequate antibiotic therapy was 41.0 hours [IQR 5.8-59.5] (n = 75). The case-fatality rate was not different among those that received adequate compared to inadequate therapy by 48 hours as compared to inadequate empiric therapy (9/42; 21% vs. 7/37; 19%; p = 1.0).

**Conclusion:**

Inadequate empiric therapy is common among patients with EK-ESBL bacteremia in our region but was not associated with adverse mortality outcome.

## Introduction

Organisms elaborating extended-spectrum beta-lactamases (ESBL) are now found worldwide [1]. Bloodstream infections caused by these microbes are increasing in the community [2,3] and in hospitals [4-6]. These organisms are typically multi-drug resistant [7] and the risk of inadequate empiric therapy [8-11] while awaiting susceptibility results is high. ESBL-bacteremias are also a risk factor for adverse outcome [10,11]. Previous studies have typically been conducted in highly selected populations including specific age ranges, location of acquisition (i.e. community or hospital). Furthermore, these infections have typically been assessed in tertiary care referral centres. These results may not reflect practices in populations at large and generalization elsewhere may be limited.

Few studies have included a broad range of ages, location of acquisition, and multiple centres, and none have been from Canada. It was therefore of interest to describe the empiric therapy of bacteremias caused by *Escherichia coli *and *Klebsiella pneumonia *elaborating ESBL enzymes (EK-ESBL) in a non-selected population of patients in a large Canadian health region.

## Methods

### Study population

Prior to a recent restructuring as Alberta Health Services, The Calgary Health Region (CHR) administered virtually all medical and surgical care to the residents of the cities of Calgary and Airdrie and a large surrounding area (population 1.2 million) in the Province of Alberta, Canada. All patients with bacteremias caused by EK-ESBL organisms from January 1, 2000 to December 31, 2007 in the CHR were included.

### Study protocol

An active, surveillance cohort design was utilized. As previously described, surveillance for EK-ESBL bacteremias was conducted by Calgary Laboratory Services [12]. International Statistical Classification of Disease and Health Related Problems (ICD) codes were translated into Charlson Comorbidity Scores using standardized algorithms [13-15] and an online macro [16]. All antimicrobial therapies prescribed were determined using the regional pharmacy database [17]. EK-ESBL infections were defined by the isolation of these organisms from one or more sets of aseptically obtained blood culture bottles. The presence or absence of ESBL-producing *E. coli *or *K. pneumoniae *cultures obtained from non-blood sites within ± 48 hours of the index incident blood culture draw was also assessed. Repeated isolation from blood within 365 days was considered to be the same incident infection and thereafter as new episodes.

Empiric therapy was defined as that therapy initiated prior to the availability of blood culture results and sensitivities. Time to first therapy was defined as the time from blood culture draw to receipt of the first dose of antibiotics. Adequate therapy was defined by the receipt of a standard parenteral dose of an antimicrobial to which the organism was fully susceptible *in vitro *based on Clinical and Laboratory Standards Institute (CLSI) breakpoints or a standard oral dose of an antimicrobial with high bioavailability by this route, to which the organism was also fully susceptible. ESBL-producing organisms were assumed to be resistant to cefazolin, ceftriaxone, ceftazidime, aztreonam, and cefipime. If the patient was given adequate antibiotics before or at the time of the blood culture draw then the time to first adequate therapy was recorded as zero hours. Definitions of nosocomial, healthcare associated and community-acquired infections have been previously described [12].

### Laboratory Methods

Clinical isolates were cultured, speciated, and tested for antimicrobial susceptibility by standard techniques [18]. At Calgary Laboratory Services, all Enterobacteriaceae isolates are routinely screened for ESBL production. Minimum inhibitory concentrations (MICs) were determined by Vitek 2™ (Vitek AMS; bioMérieux Vitek Systems Inc., Hazelwood, MO). The quality control strains used for this part of the study were *E. coli *ATCC 25922, *E. coli *ATCC 35218 and *Pseudomonas aeruginosa *ATCC 27853. The presence of ESBLs was detected in clinical isolates of *E. coli *by using the CLSI criteria for ESBL screening and disk confirmation tests [18]. Disks for ESBL confirmation tests were obtained from Oxoid Inc. (Nepean, Ontario, Canada). *K. pneumoniae *ATCC 700603 and *E. coli *ATCC 25922 were used as positive and negative controls, respectively. Isoelectric focusing (IEF) which included cefotaxime hydrolysis and inhibitor profiles in polyacrylamide gels was performed on freeze-thaw extracts as previously described [19]. Polymerase chain reaction (PCR) amplification for *bla*_*CTX*-*Ms*_, *bla*_*OXAs*_, *bla*_*TEMs*_, *bla*_*SHV*_, was carried out on the isolates with a GeneAmp 9700 ThermoCycler instrument (Applied Biosystems, Norwalk, Con) using PCR conditions and primers as previously described [19,20]. Automated sequencing was performed on the PCR products with the ABI Prism 3100 Genetic analyzer (Applied Bio-systems, Norwalk, Con) as previously described [21,22].

### Statistical analysis

Analysis was performed using Stata version 10.0 (Stata Corp, College Station, TX). Non-normally distributed data were described using medians with interquartile ranges (IQR) and compared using the Mann-Whitney test. Categorical data were compared using the Fisher's Exact or chi^2 ^tests. P-values less than 0.05 were considered significant and those between 0.05 and 0.1 were considered to represent a trend.

## Results

### Population Characteristics

During the study period, 90 episodes of EK-ESBL bacteremias were identified. Detailed treatment and outcome data were available for analysis in 79 (88%) episodes occurring among 76 patients. Forty-four (56%) episodes occurred in males and the median age was 70 (IQR 60.6-70.1) years. The median crude Charlson Comorbidity Score was 2 (IQR 0-3) and the median age-adjusted score was 4 (IQR 2-6). Twenty-four (30%) of the bacteremias were nosocomial; 33 (42%) were healthcare associated and 22 (28%) were community acquired. The most common comorbid conditions were renal disease, n = 19 (24%); malignancy, n = 16 (20%); diabetes without complications, n = 9 (11%), and diabetes with complications, n = 10 (13%) as shown in Table 1.

**Table 1:** Characteristics of the population.

FACTOR	CASE FATALITY WITH FACTOR	CASE FATALITY WITHOUT FACTOR	RELATIVE RISK (95% CI)		P-VALUE
Male	6/44 (14%)	10/35 (29%)	0.48 (0.19-1.19)		0.09

Charlson ≥ median(4) (Age Adjusted)	12/45 (27%)	4/34 (12%)	2.27 (0.80-6.41)		0.09

Cerebrovascular Disease	1/3 (33%)	15/76 (20%)	1.69 (0.32-8.91)		NS

Congestive Heart Failure	1/7 (14%)	15/72 (21%)	0.69 (0.11-4.45)		NS

Chronic Pulmonary Disease	0/1 (0%)	16/78 (21%)	0.00()		NS

Dementia	1/4 (25%)	15/75 (20%)	1.25 (0.22-7.24)		NS

Diabetes without complications	0/9 (0%)	16/70 (23%)	0.00()		NS

Diabetes with complications	1/10 (10%)	15/69 (22%)	0.46 (0.07-3.11)		NS

Paraplegia	0/1 (0%)	16/78 (21%)	0.00()		NS

Mild Liver Disease	3/4 (75%)	13/75 (17%)	4.32 (2.04-9.17)		0.03

Severe Liver Disease	2/4 (50%)	14/75 (19%)	2.68 (0.90-7.95)		NS

Malignancy	5/21 (24%)	11/58 (19%)	1.25 (0.49-3.19)		NS

Metastatic	0/1 (0%)	16/78 (21%)	0.00()		NS

Myocardial Infarction	1/3 (33%)	15/76 (20%)	1.69 (0.32-8.91)		NS

Peripheral Vascular Disease	0/1 (0%)	16/78 (21%)	0.00()		NS

Peptic Ulcer Disease	0/2 (0%)	16/77 (21%)	0.00()		NS

Rheumatologic	2/4 (50%)	14/75 (19%)	2.68 (0.90-7.95)		NS

Renal	5/19 (26%)	11/60 (18%)	1.43 (0.57-3.61)		NS

Dialysis	2/5 (40%)	14/74 (19%)	2.11 (0.65-6.83)		NS

### Microbiological Characteristics

Of the 79 bacteremic episodes 72 (91%) were *E. coli *and 7 (9%) were *K. pneumoniae*. One *Klebsiella pneumoniae *bacteremia was polymicrobial with *Pseudomonas aeruginosa*. One *Escherichia coli *bacteremia was polymicrobial with a viridans group streptococcus. Neither of these second organisms affected adequacy of antimicrobial therapy as they were sensitive to all major classes of antibiotics. Thirty-nine bacteremias (49%) were primary; 38 (48%) were from a urinary source; 2 (3%) resulted from pneumonias and 1 (1%) patient had a biliary source. One patient had two sources documented (one urinary and the other biliary). Although typing was not performed for *K. pneumoniae *isolates, *E. coli *ESBL's were typed as 1 CTX-M-2, 1 CTX-M-3, 27 CTX-M-14, 35 CTX-M-15, 5 SHV-2, 1 TEM-52 and 1 was unknown.

### Description of therapy

The median time to initial empiric therapy was 4.9 (IQR 0-13.9) hours and the median time to first adequate antibiotic therapy was 41 (IQR 5.8-59.5) hours. Seventy-four (94%) of episodes had received empiric antibiotic therapy by 48 hours, but only 42 (53%) were prescribed an adequate antibiotic regimen by this time point. Specific details of empiric therapy and initial antibiotics chosen are described in Table 2 and the adequacy of therapy and first adequate antibiotics utilized are shown in Table 3.

**Table 2:** Description of empiric therapy *.

FACTOR	CASE FATALITY WITH FACTOR	CASE FATALITY WITHOUT FACTOR	RELATIVE RISK (95% CI)	P-VALUE
Empiric by t = 0	7/29 (24%)	9/50 (18%)	1.34 (0.56-3.22)	NS

Empiric by t = 8 hours	12/48 (25%)	4/31 (13%)	1.94 (0.69-5.47)	NS

Empiric by t = 24 hours	14/68 (21%)	2/11 (18%)	1.13 (0.30-4.32)	NS

Empiric by t = 48 hours	15/74 (20%)	1/5 (20%)	1.01 (0.17-6.20)	NS

Empiric at t > 48 hours	0/3 (0%)	16/76 (21%)	0.00	NS

Never received empiric therapy.	0/1 (0%)	16/78 (21%)	...	...

*Empiric Treatment Class*				

Carbapenem	0/10 (0%)	16/69 (24%)	0.00 ()	0.09

Aminoglycoside	1/7 (14%)	15/72 (21%)	0.67 (0.10-4.41)	NS

Fluoroquinolone	3/13 (23%)	13/66 (20%)	1.17 (0.39-3.54)	NS

Beta-lactam/Beta-lactamase inhibitor combination	6/16 (38%)	10/63 (16%)	2.36 (1.01-5.53)	0.06

Cephalosporin	5/30 (17%)	11/49 (22%)	0.74 (0.29-1.93)	NS

Beta-lactam	0/5 (0%)	16/74 (22%)	0.00 ()	NS

* Empiric therapy was defined as that therapy initiated prior to the availability of blood culture results and sensitivities.

**Table 3:** Description of adequacy of therapy **.

FACTOR	CASE FATALITY WITH FACTOR	CASE FATALITY WITHOUT FACTOR	RELATIVE RISK (95% CI)	P-VALUE
Adequate by t = 0	1/11 (9%)	15/68 (22%)	0.41 (0.06-2.81)	NS

Adequate by t = 8 hours	6/22 (27%)	10/57(18%)	1.55 (0.64-3.76)	0.09

Adequate by t = 24 hours	7/28 (25%)	9/51 (18%)	1.42 (0.59-3.39)	0.10

Adequate by t = 48 hours	9/42 (21%)	7/37 (18%)	1.13 (0.47-2.74)	NS

Adequate at t > 48 hours	3/33 (9%)	13/46 (28%)	0.32 (0.10-1.04)	NS

Never received adequate therapy	4/4 (100%)	12/75 (16%)	6.25 (3.72-10.5)	0.01

*First Adequate Antibiotic Choice*				

Beta-lactam/Beta-lactamase inhibitor combination	6/28 (21%)	10/51 (20%)	1.09 (0.44-2.69)	NS

Carbapenem	4/30 (13%)	12/49 (24%)	0.54 (0.19-1.53)	NS

Fluoroquinolone	1/4 (25%)	15/75 (20%)	1.25 (0.22-7.24)	NS

Aminoglycoside	1/10 (10%)	15/69 (22%)	0.46 (0.07-3.11)	NS

Sulfa	1/3 (33%)	15/76 (20%)	1.69 (0.32-8.91)	NS

** Adequate therapy was defined by the receipt of a standard parenteral dose of an antimicrobial to which the organism was fully susceptible *in vitro *based on Clinical and Laboratory Standards Institute (CLSI) breakpoints or a standard oral dose of an antimicrobial with high bioavailability by this route, to which the organism was also fully susceptible.

### Outcomes

Overall 63 cases (80%) survived to discharge. The case-fatality rate associated with an episode of *E. coli *bacteremia was 22% (16/72) whereas this was 0% (0/7) for *K. pneumoniae*. Those who received adequate therapy by 8 hours (6/22; 27% vs. 6/53; 11%; p = 0.088) and 24 hours (7/28; 25% vs. 5/42; 12%; p = 0.096) trended towards a higher likelihood of death. However, there was no significant difference in mortality when comparing those patients who were adequately treated to those inadequately treated by 48 hours (9/42; 21% vs. 7/37; 19%; p = 1.0). Statistical trends toward a worse clinical outcome was associated with the following factors: age-adjusted Charlson score greater than the median (12/45; 27% vs. 4/34; 12% mortality; p = 0.087), female gender (10/35; 28.5% vs. 6/38; 13.6% mortality; p = 0.087); and having been treated empirically with a beta-lactam/beta-lactamase inhibitor combination (6/16; 38% vs. 10/53; 18% mortality; p = 0.063). There was no association between ESBL type and mortality. Of 10 patients empirically treated with a carbapenem none died vs. 16/53 (30%) patients having died who were not empirically treated with a carbapenem (p = 0.089). Of note empiric cephalosporin therapy was not associated with a worse outcome (5/30; 16% vs. 11/49; 22%, p = 0.375). The case-fatality rate was highest for patients with nosocomial infections (38%; 9/24), followed by those with healthcare associated infections (15%; 5/33) and community-acquired infections (9%; 2/22 patients); p = 0.046.

## Discussion

This study describes the contemporary treatment of ESBL-producing *E. coli *and *K. pneumoniae *in a large Canadian health region. Although we found high rates of inadequacy of therapy, we did not observe an increased risk for death associated with inadequate therapy.

It should be noted that our data was also analyzed using only patients with *E. coli *bacteremia as *K. pneumoniae *represented such a small proportion of the overall cohort (data not shown). The major conclusions were unchanged. The small number of *K. pneumoniae *isolates included prohibits generalization to bacteremias caused by this organism.

It is a curious finding that when comparing those who received adequate therapy by 8 and 24 hours to those that were inadequately treated that there was a trend in favour of inappropriate therapy. It is widely accepted that early appropriate antibiotic therapy should be protective [23,24]. We speculate that this observation may be explained by sicker patients (who are at higher risk for death) may be more likely to get broader and more prompt empiric antimicrobial as a result of their clinical condition. However, it is a limitation of our study that we did not collect data to measure severity of illness to confirm this suspicion. The possibility also exists that this was a spurious finding, as the difference was no longer observed by 48 hours of observation. It must be recognised that our study involved a relatively small cohort of patients and was underpowered to detect a clinically significant difference in outcome.

In our study the most common type of ESBL enzyme is the CTX-M type, specifically in our region CTX-M-14 and CTX-M-15. CTX-M type enzymes in general and CTX-M-15 in particular have been shown in previous literature [1] to be the most widely disseminated ESBL enzymes. Our study reaffirms these finding. More studies are needed to determine why these ESBL types are more prevalent than others.

Piperacillin/tazobactam is the most commonly used broad-spectrum antibiotic in our region (data not shown). Our study suggests a poorer prognosis experienced by patients treated with this agent and this has been previously been demonstrated by others [25,26]. It is notable that 5 of 6 patients treated with piperacillin/tazobactam who died were sensitive by CLSI (< = 16 mg/L) and EUCAST (< = 8 mg/L) breakpoints. These data suggest that piperacillin/tazobactam should not be used for serious infections due to EK-ESBL even in the setting of *in vitro *susceptibility. Most authors agree that carbapenems are the drugs of choice [26-29] for serious infections caused by organisms that elaborate ESBLs. Our observation that all 10 patients who received empiric carbapenem therapy survived, while not proving, supports this principle. Overall EK-ESBL bacteremias are relatively uncommon. The challenge to researchers and clinicians is to select patients appropriately for early broad-spectrum therapy in an attempt to improve outcome while minimizing over treatment and the associated risks of anti-microbial resistance.

## Competing interests

The authors declare that they have no competing interests.

## Authors' contributions

VC performed the primary data analysis and manuscript draft. JDDP performed the molecular typing of ESBL enzymes. BD and TR contributed to data collection and database management.

DLC and DBG contributed to study design and data collection. KL contributed to study conception and design, data collection, analysis, and manuscript drafting. All authors critically reviewed and approved the manuscript.
